# Authors’ Response to Peer Reviews of “Machine Learning–Based Hyperglycemia Prediction: Enhancing Risk Assessment in a Cohort of Undiagnosed Individuals”

**DOI:** 10.2196/60174

**Published:** 2024-09-11

**Authors:** Kolapo Oyebola, Funmilayo Ligali, Afolabi Owoloye, Blessing Erinwusi, Yetunde Alo, Adesola Z Musa, Oluwagbemiga Aina, Babatunde Salako

**Affiliations:** 1 Nigerian Institute of Medical Research, Lagos, Nigeria; 2 Centre for Genomic Research in Biomedicine, Mountain Top University, Ibafo, Nigeria

**Keywords:** hyperglycemia, diabetes, machine learning, hypertension, random forest

*This is the authors’ response to peer-review reports for “Machine Learning–Based Hyperglycemia Prediction: Enhancing Risk Assessment in a Cohort of Undiagnosed Individuals.”*

## Round 1 Review

### Reviewer K [1]

1. *In this paper [**2**], describe dataset features in more detail and its total size and size (train/test) as a table.*

Response: The comprehensive list of the dataset features and size are described in Additional File 2, which has now been added to the revised submission. A description of the train/test ratio is available in the Supplementary Methods section of Additional File 2.

2. *Pseudocode/flowchart and algorithm steps need to be inserted.*

Response: The flowchart/pipeline for the algorithm development was described in Figure 1, while the link to the GitHub page describing the pseudocode has been attached on page 6 of the manuscript document.

3. *Time spent needs to be measured in the experimental results.*

Response: A column has been added to Table 1 to define the time taken for each of the model classifiers

4. *Limitation and Discussion sections need to be inserted.*

Response: These sections have now been inserted.

5. *All metrics need to be calculated such as precision, recall, and receiver operating characteristic curves, in the experimental results.*

Response: Metrics have been provided in Table 1.

6. *The parameters used for the analysis must be provided in a table.*

Response: The parameters have been updated in Table 1.

7. *The architecture of the proposed model must be provided.*

Response: The architecture of the proposed (random forest) model has been described in the last paragraph (page 11) of the manuscript body.

8. *The authors need to make a clear proofread to avoid grammatical mistakes and typo errors.*

Response: We have carefully reread the manuscript and corrected all identified errors.

9. *Add future work in last section (conclusion), if any.*

Response: We have updated the manuscript to include some statements on future work. Please see pages 13 and 14 of the manuscript file.

10. *The authors need to add recent articles in related work and update them.*

Response: We have added three more citations of machine learning (ML)–related articles published by JMIR Publications on blood glucose prediction.

11. *To improve the Related Work and Introduction sections, authors are recommended to review these highly related research work papers:*

- *El-Hafeez TA, Shams MY, Elshaier YAMM, Farghaly HM, Hassanien AE. Harnessing machine learning to find synergistic combinations for FDA-approved cancer drugs.* Sci Rep*. Jan 29, 2024;14(1):2428. [doi: 10.1038/s41598-024-52814-w] [Medline: 38287066]*
- *Hassan E, El-Hafeez TA, Shams MY. Optimizing classification of diseases through language model analysis of symptoms.* Sci Rep*. Jan 17, 2024;14(1):1507. [doi: 10.1038/s41598-024-51615-5] [Medline: 38233458]*
- *Omar A, El-Hafeez TA. Optimizing epileptic seizure recognition performance with feature scaling and dropout layers.* Neural Computing Applications*. Nov 24, 2024;36:2835-2852. [doi: 10.1007/s00521-023-09204-6]*
- *Hady DAA, El-Hafeez TA. Predicting female pelvic tilt and lumbar angle using machine learning in case of urinary incontinence and sexual dysfunction.* Sci Rep*. Oct 20, 2023;13(1):17940. [doi: 10.1038/s41598-023-44964-0] [Medline: 37863988]*
- *Eliwa EHI, El Koshiry AM, El-Hafeez TA, Farghaly HM. Utilizing convolutional neural networks to classify monkeypox skin lesions.* Sci Rep*. Sep 3, 2023;13(1):14495. [doi: 10.1038/s41598-023-41545-z] [Medline: 37661211]*
- *Farghaly HM, Shams MY, El-Hafeez TA. Hepatitis C Virus prediction based on machine learning framework: a real-world case study in Egypt.* Knowledge Inf Syst*. Mar 2, 2023;65:2595-2617. [doi: 10.1007/s10115-023-01851-4]*

Response: The suggested articles have been reviewed and cited in the manuscript accordingly.

### Reviewer V [3]

#### General Comments

*This paper introduces an ML methodology for predicting hyperglycemia in one of the cohorts taken from a suburban Nigerian region. The authors present the details of the methodology for participant recruitment and screening, data analysis, and selection of ML models.*

Response: We appreciate the reviewer for meticulously evaluating our manuscript and providing important suggestions to improve the quality of the article. We have carefully revised the manuscript to address the comments and incorporate their suggestions.

#### Specific Comments

##### Major Comments

1. *The introduction and motivation behind the work are well written. However, there is not enough literature done on the ML aspect of noncommunicable disease prediction; please also cite some of the recent work where ML-based methods are used for noncommunicable disease prediction.*

Response: We have cited eight more studies on ML-based noncommunicable disease prediction.

2. *Before selecting the features, was there any domain expert consulted? If yes, please provide reasoning on some aspect of feature selection.*

Response: We considered domain knowledge and input from the study clinicians to guide our feature selection.

3. *How were the different ML models selected for the experiment? Please elaborate on some selection criteria such as the combination of tree-based models with other ensemble approaches such as random forest.*

Response: We used a Python library, LazyPredict, to automate the selection and performance assessment of the algorithms LazyPredict supports a wide range of supervised learning algorithms of which random forest emerged as the top-performing algorithm in this case, consistently delivering the highest accuracy among the tested models.

##### Minor Comments

1. *In Table 2, please reduce the decimal precision up to 2 digits.*

Response: We have now edited the table values into 2 decimal places.

2. *Figure 1 could be improved with a flow diagram to provide better readability and details of each step.*

Response: We have reproduced Figure 1 into a flow diagram.

### Reviewer AD [4]

#### General Comments

*Overall strong paper! This was an interesting study on the use of ML to predict hyperglycemia in a cohort of undiagnosed individuals from Nigeria. I feel like this work is a strong contribution to the field of public health, especially within the context of noncommunicable diseases in developing countries. I also like that it is backed well with quantitative methods. The strengths of this manuscript lie in its detailed methodology and its comprehensive data analysis.*

Response: We appreciate the reviewer for meticulously evaluating our manuscript and providing important suggestions to improve the quality of the article. We have carefully revised the manuscript to address the comments and incorporate their suggestions.

#### Specific Comments

##### Major Comments

- *While the study demonstrates a robust analytical approach, it would benefit from external validation with an independent dataset. This would strengthen the findings and ensure the model’s generalizability and applicability in different populations.*

Response: We appreciate and agree with the reviewer on this useful insight. It is, in fact, the next phase of our model development pipeline as we plan to evaluate the sensitivity, specificity, positive and negative predictive values, and overall accuracy to determine the model’s ability to correctly detect real-life cases of hyperglycemia compared with the traditional detection tools. We are drafting this proposal for grant funding, and hopefully, we will be able to address this aspect. However, the analytical approach adopted in this manuscript defines the entire scope of the present study.

- *The manuscript could be improved by providing more context on the selection of the ML algorithms used in the study. An explanation of why certain algorithms were chosen and others potentially excluded would offer clarity.*

Response: We used a Python library, LazyPredict, to automate the selection and performance assessment of the algorithms. LazyPredict supports a wide range of supervised learning algorithms, of which random forest emerged as the top-performing algorithm in this case, consistently delivering the highest accuracy among the tested models. This has been elucidated in the Methodology section.

##### Minor Comments

- *The manuscript occasionally uses technical jargon that might not be easily understandable to readers not familiar with ML. Simplifying the language or providing brief explanations will make the paper more accessible.*

Response: We have simplified the content of the manuscript to enhance readability.

- *The study’s potential for real-world application would be clearer with a section on future work, detailing how these algorithms could be deployed in clinical settings or used in larger-scale studies (I can see how this might be a tangential research direction, but this would still be great given the potential impact).*

Response: We have now elucidated the future plans for real-world application under the Limitation and Future Direction section.
